# The roles of the zinc finger transcription factors XlnR, ClrA and ClrB in the breakdown of lignocellulose by *Aspergillus niger*

**DOI:** 10.1186/s13568-016-0177-0

**Published:** 2016-01-16

**Authors:** Roxane Raulo, Matthew Kokolski, David B. Archer

**Affiliations:** School of Life Sciences, University of Nottingham, University Park, Nottingham, NG7 2RD UK

**Keywords:** Glysosyl-hydrolases, Regulation, Transcription factor, pH, Cell wall

## Abstract

Genes encoding the key transcription factors (TF) XlnR, ClrA and ClrB were deleted from *Aspergillus niger* and the resulting strains were assessed for growth on glucose and wheat straw, transcription of genes encoding glycosyl hydrolases and saccharification activity. Growth of all mutant strains, based in straw on measurement of pH and assay of glucosamine, was impaired in relation to the wild-type (WT) strain although deletion of *clrA* had less effect than deletion of *xlnR* or *clrB*. Release of sugars from wheat straw was also lowered when culture filtrates from TF deletion strains were compared with WT culture filtrates. Transcript levels of *cbhA*, *eglC* and *xynA* were measured in all strains in glucose and wheat straw media in batch culture with and without pH control. Transcript levels from *cbhA* and *eglC* were lowered in all mutant strains compared to WT although the impact of deleting *clrA* was not pronounced with expression of *eglC* and had no effect on *xynA*. The impact on transcription was not related to changes in pH. In addition to impaired growth on wheat straw, the *ΔxlnR* strain was sensitive to oxidative stress and displayed cell wall defects in the glucose condition suggesting additional roles for XlnR. The characterisation of TFs, such as ClrB, provides new areas of improvement for industrial processes for production of second generation biofuels.

**Electronic supplementary material:**

The online version of this article (doi:10.1186/s13568-016-0177-0) contains supplementary material, which is available to authorized users.

## Introduction

Enzymes from filamentous fungi have a key role in the degradation of the most abundant biopolymers found in nature, cellulose and hemicelluloses. For this reason, these enzymes are of great interest in the industrial conversion of lignocellulosic substrates into component sugars which then serve as substrates for the synthesis of biofuels and other products. Fungi are the predominant source of enzymes currently being used on an industrial scale for this purpose (Archer 2000; Gusakov 2011; Sims et al. 2010). The production of plant cell wall-degrading enzymes is mainly regulated at the transcriptional level in filamentous fungi but insufficient is known about the transcription factors (TFs) involved in this regulation. The mechanisms of regulation of genes encoding cellulases and hemicellulases in filamentous fungi have been studied, in *Aspergillus* spp. (Noguchi et al. 2009; van Peij et al. 1998a), *Trichoderma* spp. (Stricker et al. 2008) and *Neurospora crassa* (Coradetti et al. 2012) amongst other species. The production of many of these enzymes is coordinately regulated, and they are induced in the presence of the substrate polymers. Molecules such as gentiobiose in *Penicillium* spp. (Kurasawa et al. 1992) or xylose in *A. niger* (Mach-Aigner et al. 2012) and sophorose in *T. reesei* (Sternberg and Mandels 1979) have been shown to be inducers of genes encoding cellulases and hemicellulases.

The family of zinc finger TFs is one of the most important families of TFs, and they have regulatory functions in development and metabolism (Caddick and Dobson 2008). Sequencing of the *A. niger* CBS 513.88 genome (Pel et al. 2007) has predicted that the genome encodes 286 TFs containing the Zn(II)2Cys6 motif. Nevertheless, the majority of these TFs have not been studied in detail although on-going programmes to delete the genes will facilitate their study. The xylanolytic activator XlnR is the most studied of the transcriptional activators involved in the regulation of glycoside hydrolase genes in *Aspergillus* spp. and, as Xyr1 in *T. reesei*. XlnR regulates the expression of cellulases and hemicellulases (van Peij et al. 1998a, Stricker et al. 2006, Mach-Aigner et al. 2012). Delmas et al. (2012) showed that the regulation of the expression of responsive genes in *A. niger* grown on wheat straw was sequential. Firstly, the lack of easily available carbon source led to the alleviation of CreA repression and to the induction of a subset of starvation-induced genes. A subset of Carbohydrate-Active enzyme (CAZy) (Lombard et al. 2014) encoding genes was found to be expressed upon starvation, when there is no induction by XlnR or repression by CreA. These CAZy enzymes were hypothesized to have a scouting role, to degrade carbon sources in the vicinity to release inducing sugars. These sugars can in turn induce the expression of XlnR-dependent CAZy genes. When the responses of *A. niger* to wheat straw were compared to the responses to willow, a similar pattern was observed, though there were some notable differences in expression levels (Pullan et al. 2014). Genes that had higher expression on wheat straw compared to willow included a GH62 arabinofuranosidase and two feruloyl esterases. These increases in expression could be related to compositional differences in the substrates (Pullan et al. 2014). A sequential response was also suggested in the transcriptional responses of *A. niger* to wheat straw where most of the genes encoding enzymes degrading hemicelluloses were induced earlier than genes encoding enzymes with activity towards pectins (van Munster et al. 2014). XlnR is not the only important regulator of GH-encoding genes in filamentous fungi. Among the other TFs found in Ascomycetes to be relevant to lignocellulose degradation, AraR (Arabinolytic regulator) seems to be of importance. AraR is a Zn_2_Cys_6_ TF resulting from a recent duplication event of *xlnR* and is only found in certain fungal species (Battaglia et al. 2011). Battaglia et al. (2011) showed that AraR functioned in co-operation with XlnR in the regulation of the pentose catabolic pathway. In *A. niger*, the deletions of *araR* and *xlnR* showed that the expression of CAZy-encoding genes was partly dependent on AraR. However, there was no clear evidence that some genes would be under the sole regulation of AraR (de Souza et al. 2011, 2013). Another Zn_2_Cys_6_ TF identified is ClbR (Cellobiose Response regulator) which was characterised in *Aspergillus aculeatus* (Kunitake et al. 2012). In this study, Kunitake et al. (2012) showed that ClbR regulated CAZy-encoding genes in response to cellulose and cellobiose (but not xylose) in an XlnR-dependent and independent manner. Moreover, the authors showed that the deletion of *clbR* led to a decrease of the cellobiose and cellulose responsive induction of the *cbhI*, *cmc1*, *cmc2*, *xynIb* and *xynIa* genes encoding respectively a cellobiohydrolase, two carboxymethylcellulases and two xylanases. Finally, a new regulator, ACE3 (Activator of cellulase expression 3) which contains a PFam Fungal specific TF domain, was identified in *T. reesei* (Hakkinen et al. 2014). The results of the study showed that when *ace3* was deleted or over-expressed, the expression of *xyr1* was altered, suggesting that ACE3 could be a major regulator in *T. reesei* (Hakkinen et al. 2014). In *N. crassa*, two other Zn_2_Cys_6_ family TFs CLR-1 and CLR-2 were the predominant regulators of the expression of cellulase-encoding genes (Coradetti et al. 2012). Orthologues of *clr*-*1* and *clr*-*2* have been identified in many fungal species but some of the orthologues have been demonstrated to be functionally different. *A. nidulans* ClrB has more limited functions than CLR-2 in *N. crassa* (Coradetti et al. 2012). This suggests that homologues of CLR-1 and CLR-2 could also play an important role in plant cell-wall degradation in other filamentous fungi species.

Here we characterised the roles of ClrA and ClrB in *A. niger* and further analysed the role of XlnR by phenotyping the *ΔclrA*, *ΔclrB* and *ΔxlnR* strains for growth on glucose and wheat straw substrates. We show that the regulation in *A. niger* is generally in line with other *Aspergillus* spp. but distinct from *N. crassa*.

## Materials and methods

### Fungal strains

The *A. niger* strain AB4.1 (*ΔpyrG*) (deposited as ATCC^®^ 62590™) was used in this work (van Hartingsveldt et al. 1987). The *ΔclrB* strain was constructed and kindly provided from a collaborative effort (see Acknowledgements). It was made in MA234.1 derived from MA169.4 (a derivative of AB4.1) (Carvalho et al. 2010) and contains a *hygB* marker (pGpdA-hygB-tTrpC). The AB4.1, *ΔxlnR* and *ΔclrA* strains were grown on Potato Dextrose Agar (PDA, Oxoid, UK) supplemented with 10 mM uridine (final concentration) for routine cultures. The *ΔclrB* strain was grown on PDA. Agar slopes were inoculated with *A. niger* conidia and incubated at 28 °C for 4 days with loose caps, after which time the caps were tightened and the slopes with visible conidiated growth of *A. niger* were stored at room temperature.

### Culture of *A. niger*

For cultures of *A. niger* AB4.1, *ΔxlnR*, *ΔclrA* and *ΔclrB* strains, spores were washed with 0.01 % (v/v) Tween-80 solution and 10^8^ spore, in suspension were inoculated into 250 ml sterile Erlenmeyer flasks containing 100 ml AMM (Delmas et al. 2012) containing 1 % (w/v) glucose or ball-milled wheat straw (1 % w/v) (Delmas et al. 2012) and incubated for 48 h at 28 °C on a shaker (New Brunswick Scientific, St Albans, UK) set to 150 rpm. Media were autoclaved at 117 °C instead of 121 °C to limit the caramelising of sugars. For studies involving time-points, the *A. niger* strain used was grown in 1 % (w/v) glucose for 48 h before being washed and transferred to AMM supplemented with 1 % (w/v) of the carbon source and incubated further for 24 or 48 h. Supernatants were collected at each time point for enzymatic analysis.

### Construction of plasmids

Molecular cloning techniques were performed using standard procedures (Shubeita et al. 1987). DNA polymerase was Phusion (NEB, Hitchin, UK). Primers were designed using the *A. niger* CBS 513.88 sequence.

### Transformation procedures

The *ΔxlnR* and *ΔclrA* strains were constructed in *A. niger* AB4.1 (*ΔpyrG*). The entire open reading frames were deleted. Strains were constructed using the method developed by Scherer and Davis (1979) based on recombination between a plasmid containing the flanking region of the gene of interest and the chromosome. As a selection/counter-selection marker we used the gene coding for the orotidine-5-phosphate decarboxylase (Boeke et al. 1984) (*pyrG*, from *Aspergillus oryzae*). After transformation of *A. niger*, cells were selected for uridine prototrophy, confirming integration of the plasmid into the chromosome. After purification of the transformants, release of the selective pressure for the integrated plasmid was achieved by propagating the clones twice on potato dextrose agar containing 10 mM uridine. Selection for cells that had excised the plasmid from the chromosome was done by plating them on media containing 4 mM of 5-fluoro-orotic acid (Melford, Ipswich, UK) and 1.6 mM uridine. Deletions of *xlnR* and *clrA* were confirmed by PCR using internal and external oligonucleotide primers and by sequencing around the respective loci. The primers used for vector construction are shown in Additional file 1: Table S1. Electroporation was used to transform *Escherichia coli*.

### RNA extraction

At each given time, mycelia were collected through Miracloth (Calbiochem, Watford, UK), washed with AMM containing no carbon source and flash frozen in liquid nitrogen prior to RNA extraction. Frozen *A. niger* mycelia were ground to a powder under liquid nitrogen using a mortar and pestle. The powders obtained for each sample were added to tubes containing 1 mL TriZol (Invitrogen, Paisley, UK) and the suspensions were incubated at room temperature for 5 min before the addition of 200 μL of chloroform. The mixtures were vortexed for 15 s and incubated at room temperature for 5 min. The two layers were separated by centrifugation at 16,060*g* for 10 min. The upper phase was then transferred to tubes containing 750 μL of isopropanol and incubated for 10 min at −20 °C. The tubes were centrifuged (16,060*g* for 10 min) before the supernatant was removed and discarded. The pellets were washed with 75 % v/v ethanol, air-dried for 2–5 min and then re-dissolved in 100 μl DEPC-treated H_2_O for 20 min at 4 °C. All solutions, unless otherwise stated, were prepared in DEPC-treated H_2_O. The RNA was purified again after extraction using a NucleoSpin RNA II kit (Macherey–Nagel, Düren, Germany) including the additional on-column DNAse digestion according to the manufacturer’s protocol to eliminate genomic DNA contamination.

### cDNA amplification by RT-PCR and qRT-PCR

cDNA was synthesised from the isolated RNA by reverse transcription using SuperScript III Reverse Transcriptase (Invitrogen, Paisley, UK) according to the manufacturer’s protocol. Oligo (dT) was used as a primer on 0.5 μg of RNA per reverse transcription reaction. The cDNA was checked by PCR using Phusion polymerase (NEB, Hitchin, UK) with 0.5 μl of cDNA in a 20 μl PCR. The PCR program used had an initial denaturation step at 98 °C for 2 min. After the initial denaturation the program continued with 30 cycles of denaturation at 98 °C for 20 s, hybridisation at 60 °C for 20 s, and elongation at 72 °C for 20 s. The final extension was at 72 °C for 5 min. qRT-PCR amplifications were carried out using the Applied Biosystems 7500 Fast Real-Time PCR system. qPCR reactions were prepared in a total volume of 10 μl using the 2X FAST SYBR-Green Master Mix with 0.25 μl of cDNA and a final primer concentration of 0.2 μM. PCRs were carried out for 40 cycles; denaturation at 95 °C for 15 s, annealing at 67 °C for 30 s, and extension at 60 °C for 60 s. The specificity of primer sets used for qRT-PCR amplification was evaluated by melting curve analysis. The Standard Curve Method was used for quantification against a known concentration of genomic DNA (Li et al. 2009). All experiments were done in biological and technical triplicates.

### Assay of reducing sugars using 3, 5-dinitrosalicylic acid (DNS)

The reaction was started by mixing 500 µL of supernatant from fungal cultures with 750 µL of 2 % wheat straw made in 50 mM citrate buffer (pH 4.8) in 2 ml tubes. The mixture was incubated overnight at 50 °C under agitation at 250 rpm. The reaction was stopped by heating the samples at 100 °C. The samples were centrifuged for 10 min at 16,060*g* and the supernatants were transferred to a new tube. The amount of reducing sugars liberated in the enzyme reaction was assayed by mixing 80 μL of the enzyme reaction with 120 μL DNS solution in a 96-well PCR microplate, heating to the boiling point for 5 min and cooling on ice for 5 min. 150 μL of each sample were transferred to a new 96-well microplate before measuring the absorbance at 540 nm using an ELISA micro-plate reader (Multiskan™ GO Microplate Spectrophotometer, Thermo Scientific, Loughborough, UK). Ranges of glucose standards run at the same time were used to calculate the saccharification activity of the samples. All experiments were done in biological and technical triplicates.

### Glucosamine assay and measurement of pH

The contents of the flasks were filtered and freeze-dried in 50 ml centrifuge tubes and included a wheat straw only control. Samples (0.1 g dry weight for the glucose cultures and the full content of the flask for the wheat straw cultures) were hydrolyzed by 2 ml of concentrated sulphuric acid (98 %) at room temperature for 24 h. Similarly, N-acetylglucosamine standards were placed into 50 ml centrifuge tubes and 2 ml of concentrated sulphuric acid (98 %) was added at room temperature for 24 h. The mixtures were transferred into 100 ml bottles with 35 ml of dH_2_O already inside and then autoclaved at 121 °C for 15 min. After cooling, the solution was neutralized with NaOH to pH 7 and further diluted to 100 ml with dH_2_O. 1 ml of the above sample solutions was transferred into a 2 ml tube. 1 ml acetyl acetone reagent (4 % (v/v) acetyl acetone in 1.25 N Na_2_CO_3_) was added and then incubated at 100 °C for 20 min. The samples were transferred into a 15 ml centrifuge tube. After cooling to room temperature, 6 ml of absolute ethanol was added and mixed gently. 1 ml of Ehrlich reagent (1.6 g of N–N dimethyl-*p*-aminobenzaldehyde in 60 ml solution containing 50:50 (v/v) of absolute ethanol: concentrated HCl) was added and mixed gently. The mix was incubated at 65 °C for 10 min and the absorbance value was determined at 530 nm. The glucosamine value was calculated back from the absorbance measurement using a standard curve of known concentrations of N-acetylglucosamine (Sigma-Aldrich, Dorset, UK). The glucosamine content of a known dry weight sample was assayed in both glucose and wheat straw and showed no interference of the wheat straw in the measurement of glucosamine. pH was also measured using a pH 210 Microprocessor pH meter (Hanna instruments, Leighton buzzard, UK) in the cultures as a wide range of organic acids is produced by *A. niger* during fermentation which results in the acidification of the medium. Thus the measurement of pH, as an acidification of the culture medium, can serve as an indicator of growth for *A. niger* (Archer et al. 1990).

### pH-controlled experiment

A 2L fermenter (FerMac 320, Electrolab) was used for growing the cultures of AB4.1, *ΔxlnR*, *ΔclrA* and *ΔclrB* strains of *A. niger* where the pH was maintained at 5 (200 rpm, air 1L/min, 28 °C). Glucose cultures were set up as described previously in 1L sterile Erlenmeyer flasks containing 400 ml AMM, 1 % (w/v) glucose solution and incubated for 48 h at 28 °C on a shaker (New Brunswick Scientific, St Albans, UK) set to 150 rpm. The cultures were transferred to AMM supplemented with 1 % (w/v) wheat straw for the *ΔclrB* and to which was added 0.1 M uridine for the culture of the AB4.1, *ΔxlnR* and *ΔclrA* strains. Samples were taken at 6, 24 and 48 h. Supernatants were collected at each time point for enzymatic analysis.

### Cell wall composition analysis

Mycelia samples were collected as for RNA extraction and ground to a powder under liquid nitrogen. The resulting powder was washed with 5 ml of protein extraction buffer (2 % SDS, 40 mM β-mercaptoethanol, 50 mM Tris–HCl, 5 mM EDTA, pH 7.4) and incubated at room temperature for 10 min before being centrifuged in a PK121R multispeed centrifuge (ALC) swing-out rotor for 10 min at 4000*g*. The pellets were then resuspended in 1 ml H_2_O before being freeze-dried using a ThermoSavant Modulyo^®^ Freeze-dryer coupled to a Edwards RV8 vacuum pump. 10 mg of dry cell wall mass was then resuspended in 225 µl 72 % (w/w) sulphuric acid and incubated for 3 h at room temperature with gentle shaking by hand throughout the incubation to allow complete contact of the acid solution with the polysaccharides. The samples were then further diluted to 1 M sulphuric acid by addition of 2.85 ml ultra-pure H_2_O containing 1 mg/ml fucose (internal standard) and homogenised. The suspensions were incubated 4 h at 100 °C using a boiling water bath. After incubation, the tubes were cooled to room temperature and 1 ml of the hydrolysate was transferred to a new centrifuge tube. The hydrolysate was neutralised by dropwise addition of a saturated barium hydroxide solution (40 g/L Ba(OH)_2_ in ultra-pure H_2_O) until the pH reached neutrality and incubated overnight at 4 *°*C. After incubation the pH was checked for neutrality, the volume adjusted to 10 ml with ultra-pure H_2_O and the samples were centrifuged at 4000*g* for 10 min in a PK121Rmultispeed centrifuge (ALC) swing-out rotor. 0.1 ml of the supernatant was diluted 10 times and 1 ml of the sample was taken and placed into a numbered HPLC vial for sugar analysis using the HPLC (DIONEX ICS 3000) Reagent-FreeTM Ion Chromatograph equipped with Dionex ICS-3000 system, electrochemical detection using ED 40 and computer controller. A CarboPacTM PA 20 column (3 × 150 mm) was used for the separation with a mobile phase of 10 mM NaOH and a flow rate of 0.5 mL/min. The injection volume was 10 µl and the column temperature was 30 °C.

### Fluorescence assays

1 ml of 16 h-old germinating conidia was incubated with 2′, 7′-dichlorodihydrofluorescein diacetate (H2DCF-DA, Sigma Aldrich, Dorset, UK) or Phloxine B (Sigma Aldrich, Dorset, UK) solutions (5 μM final concentration) and observations were performed under a fluorescent microscope with blue and green filter combinations. The images were taken using a GXML3201 LED microscope (Suffolk, UK) equipped with a GT Vision camera (Suffolk, UK). A 100 × objective lens and a final magnification of × 1000 were used. Microscope images were viewed and recorded using GXCapture version 7.3 software (Suffolk, UK). The fluorescence intensity was determined using the ImageJ software on 50 images taken for each condition from three biological repeats.

## Results

### The growth of the *A. nigerΔxlnR* and *ΔclrB* strains is impaired on wheat straw

**Fig. 1:**
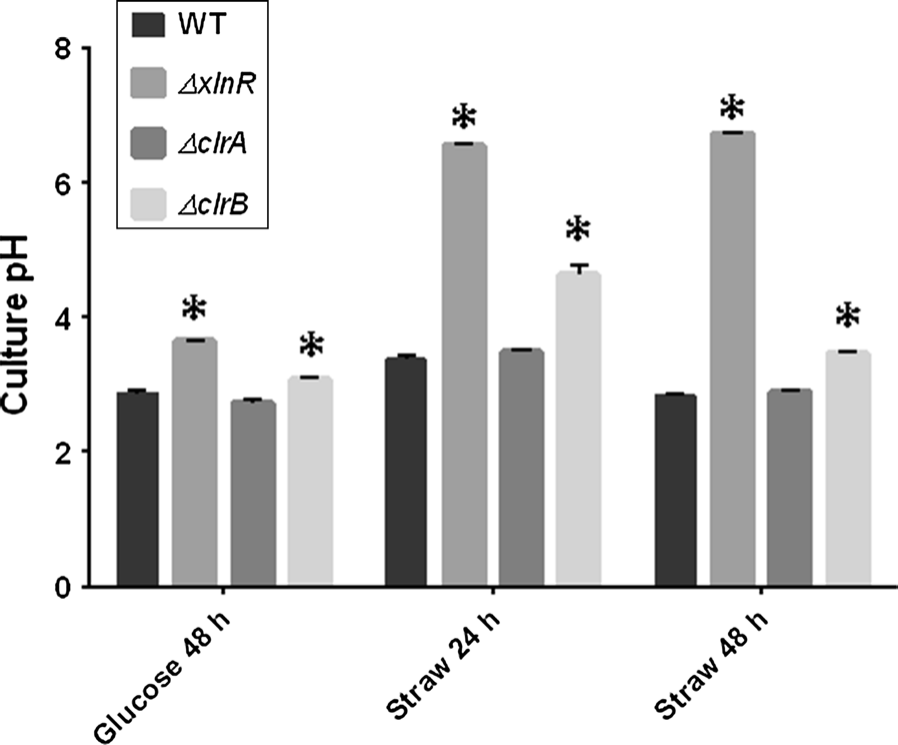
pH measurements from glucose and wheat straw cultures. For both substrates the starting pH of the medium was pH 6.5. The *results shown* represent triplicates of three independent biological samples and the *error bars* indicate the standard error of the mean. A two-way ANOVA was performed for each condition in comparison to the WT strain. * represents the conditions for which the two-way ANOVA test result had a *p*-value < 0.05 which means that the difference observed is significant when compared to the WT

The growth of the *ΔxlnR*, *ΔclrB* and *ΔclrA* strains was assayed by measuring the pH of the cultures as the strains were growing in wheat straw and by measuring the glucosamine content of the cultures. The cultures were grown for 48 h in glucose before the mycelia were transferred to wheat straw medium and after 24 and 48 h in wheat straw; the pH was measured in the cultures. For the glucose condition, after 48 h of culture, the pH had dropped from 6.5 to 2.9 for the *A. niger* WT strain and from 6.5 to 3.6 for the *ΔxlnR* strain and from 6.5 to 3.1 for the *ΔclrB* strain. In wheat straw, after transfer to the medium, the pH had dropped from 6.5 to 3.4 after 24 h and to 2.8 after 48 h for the WT strain. The pH remained at 6.5 after 24 h and increased to 6.7 after 48 h for the *ΔxlnR* strain. For the cultures of the *ΔclrB* strain, the pH decreased to 4.6 after 24 h and to 3.5 after 48 h (Fig. 1). For the three conditions tested, the data for the *ΔclrA* strain were similar to the WT strain. Statistical tests showed significant differences between the WT strain and the *ΔxlnR* and the *ΔclrB* strains in the glucose and wheat straw conditions.

**Fig. 2:**
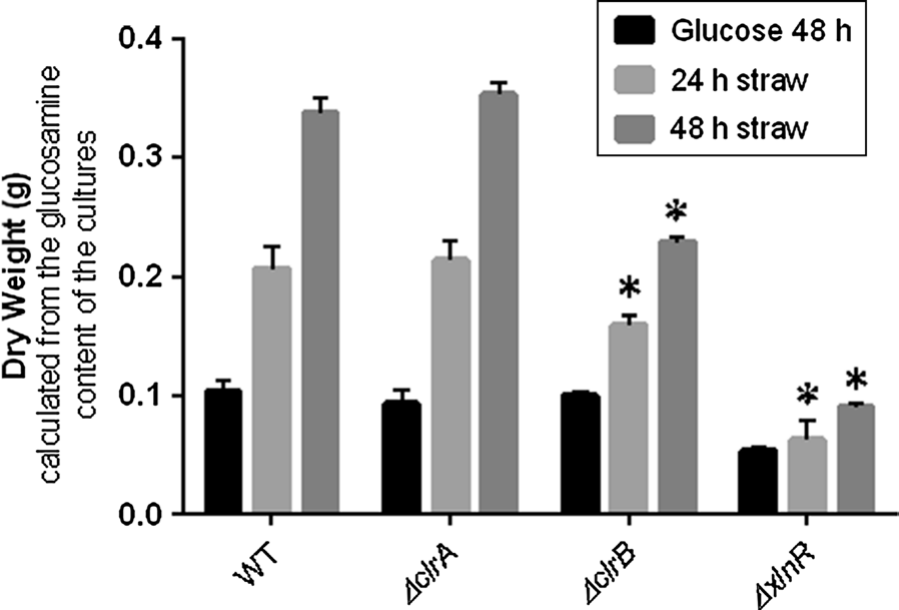
Increase in the biomass content of the *ΔxlnR*, *ΔclrB* and *ΔclrA* cultures in glucose and wheat straw. The wheat straw cultures were inoculated with 1.5 g (wet weight) of mycelium from the corresponding glucose culture. The glucosamine content was measured from 1.5 g (wet weight) for the glucose culture and from the content of the full culture for the wheat straw cultures and the dry weight was calculated back using a standard curve of the glucosamine content against the dry weight. The *results shown* represent triplicates of three independent biological samples and the *error bars* indicate the standard error of the mean. * represents the conditions for which the *t*-test result had a *p-*value < 0.05 which means that the difference observed is significant when compared to the WT

Glucosamine is a useful compound for the estimation of fungal biomass, taking advantage of the presence of chitin, poly-N-acetylglucosamine, in the cell walls of many fungi (Aidoo et al. 1981; Pusztai 1964). The measurement of the glucosamine content for the different strains was done from cultures inoculated with 1.5 g wet weight of mycelium from the corresponding 48 h glucose culture and incubated for 24 h and 48 h in wheat straw. After 48 h in glucose the amount of glucosamine in the WT strain was 3.3 mg, 1.6 mg in the *ΔxlnR* strain, 2 mg for the *ΔclrB* strain and 1.7 mg for the *ΔclrA* strain. The amounts of glucosamine after 24 h and 48 h in wheat straw were 3.5 mg and 4.7 mg respectively for the WT strain, 2.1 mg and 3 mg for the *ΔxlnR* strain, 2.3 mg and 3.4 mg for the *ΔclrB* strain and 3 and 3.9 mg for the *ΔclrA* strain (Fig. 2). Mycelial glucosamine content is about 5 % of glucosamine per g of dry mycelium (Andersen 2008) which is consistent with the value of the WT glucose time point corresponding to 3.5 % of glucosamine. The corresponding dry weights were then calculated back using a standard curve of known dry weights with their corresponding glucosamine contents.

### The supernatants of the *A. nigerΔxlnR*, *ΔclrB* and *ΔclrA* cultures contain less enzyme activity for sugar release from wheat straw

**Fig. 3:**
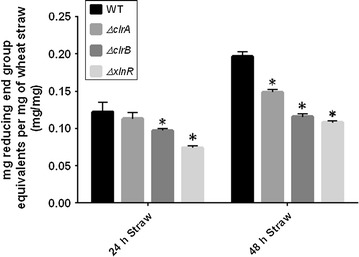
Measurement of sugars reducing ends released after incubation of equal volumes of the supernatants of the *ΔxlnR*, *ΔclrB* and *ΔclrA* wheat straw cultures with straw done by the DNS assay. A two-way ANOVA was performed for each condition in comparison to the WT strain. The *results shown* represent triplicates of three independent biological samples and the *error bars* indicate the standard error of the mean. * represents the conditions for which the two-way ANOVA test result had a *p-*value < 0.05 which means that the difference observed is significant when compared to the WT

The measurement of the enzyme activity from the supernatants of the *ΔxlnR*, *ΔclrB* and *ΔclrA* wheat straw cultures was done using the DNS assay (Miller 1959) measuring the reducing sugar ends released from the incubation of the supernatants with wheat straw. The results showed that fewer reducing sugar ends were released from the incubation of the supernatants from the three deletion strains when compared to the WT supernatant. For the WT strain, 0.12 and 0.2 mg of reducing sugars per mg of wheat straw were released from the 24 and 48 h supernatants respectively. For the deletion strains, 0.11 and 0.15 mg of reducing sugars per mg of wheat straw were released for the *ΔclrA* strain, 0.1 and 0.12 mg of reducing sugars per mg of wheat straw for the *ΔclrB* strain and 0.07 and 0.11 mg of reducing sugars per mg of wheat straw for the *ΔxlnR* strain (Fig. 3). Statistical analysis showed that the result was significantly different to the WT strain for the *xlnR* deletion strain after 24 h and the results were significantly different to the WT strain for the three deletion strains after 48 h.

### The transcript levels of the genes *cbhA*, *eglC* and *xynA* are decreased in the *A. nigerΔxlnR*, *ΔclrB* and *ΔclrA* strains and are not dependent on the pH of the culture

**Fig. 4:**
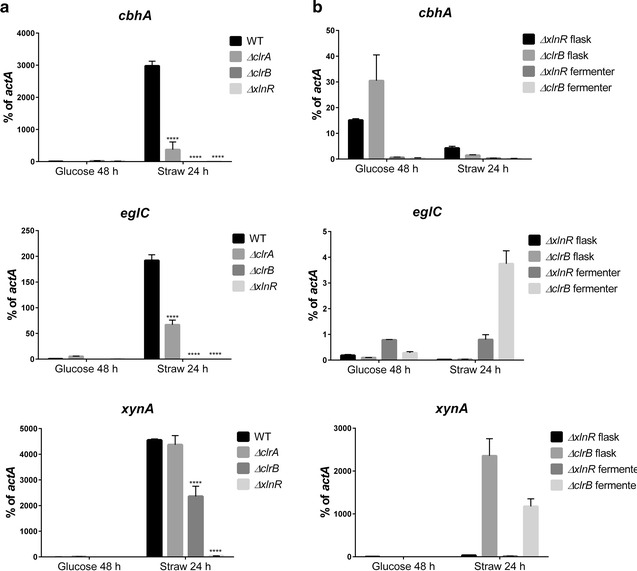
**a** Expression of the *cbhA*, *eglC* and *xynA* genes in *A. niger* grown in wheat straw media. The *results shown* represent triplicates of three independent biological samples and the *error bars* indicate the standard error of the mean. **b** Comparison of the expression of the *cbhA, xynA* and *eglC* genes in *A. niger* grown in wheat straw media under pH-controlled condition (pH 5.0 in fermenter) and no control of pH (flask). The *results shown* represent triplicates of two independent biological samples and the *error bars* indicate the standard error of the mean. For both experiments, the *expression values* represent the percentage of transcript level normalised against the level for *actA*. * represents the conditions for which the *t*-test result had a *p-*value < 0.05 which means that the difference observed is significant when compared to the WT

The transcript levels of the genes *cbhA*, *eglC* and *xynA* were measured by qRT-PCR in the *ΔxlnR*, *ΔclrB* and *ΔclrA* strains after 24 h of incubation in wheat straw. Transcripts of the genes *cbhA*, *eglC* and *xynA* were detected at very low levels in the WT strain grown on glucose, while an >100- to 1000-fold increase in transcription (% of *actA* transcript level) was measured during grown on wheat straw for the different genes (Fig. 4a). For all the mutant strains a decrease in transcription was detected on wheat straw for all the genes tested. However, for the *xynA* gene, the difference in transcript levels was not significant between the WT and the *ΔclrA* strain. The data for the *actA* gene are presented in Additional file 1: Fig. S2.

To investigate if the decrease of the transcript levels of the *cbhA* and *eglC* genes observed in the qRT-PCR experiment could be due to the non-acidification of the culture, the *ΔxlnR* and the *ΔclrB* strains were grown in a fermenter with the pH maintained at pH 5. The results showed that there was no pH effect on the transcription of the genes tested with expression levels remaining in the range of the non-controlled condition (Fig. 4b). The fold change measured for the WT strain in the fermenter condition was similar to the one measured in the non-controlled condition (data not shown).

### The transcript levels of the genes *clrA* and *clrB* are decreased in the *A. nigerΔxlnR* strain

**Fig. 5:**
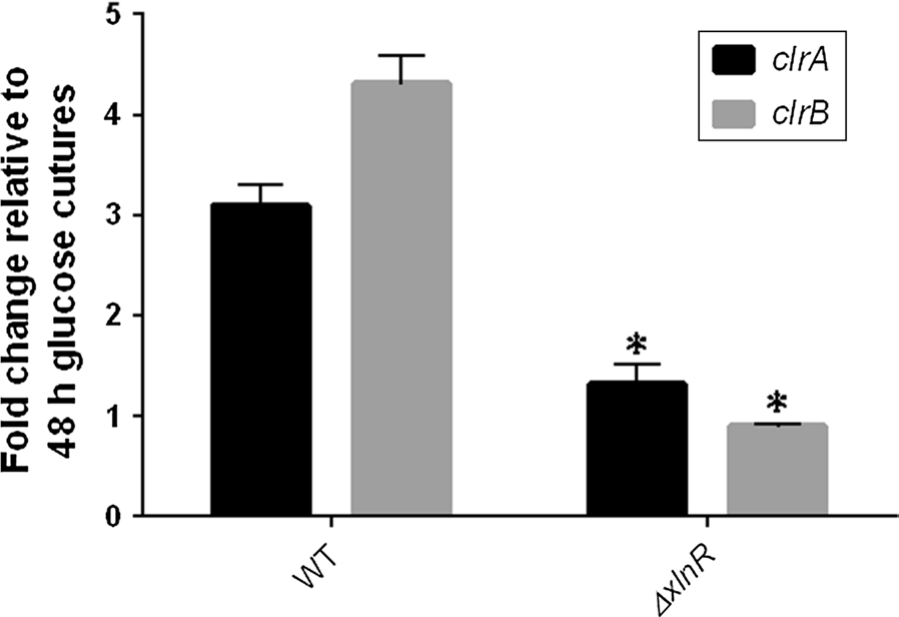
Expression of the *clrA* and *clrB* genes in *A. niger* grown in wheat straw media. The *results shown* represent triplicates of three independent biological samples and the *error bars* indicate the standard error of the mean. * represents the conditions for which the *t*-test result had a *p-*value < 0.05 which means that the difference observed is significant when compared to the WT

The expression levels of the *clrA* and *clrB* genes were measured in the WT and the *ΔxlnR* strains. The results for the WT strain showed that the *clrB* gene was more highly induced than the *clrA* gene on wheat straw. However, the transcript levels of the two genes were decreased in the *ΔxlnR* strain with around a one-fold change induction for both genes when compared to the glucose condition (Fig. 5).

### The cell wall of the *A. nigerΔxlnR* strain contains more glucosamine and less galactose than the WT strain

**Fig. 6:**
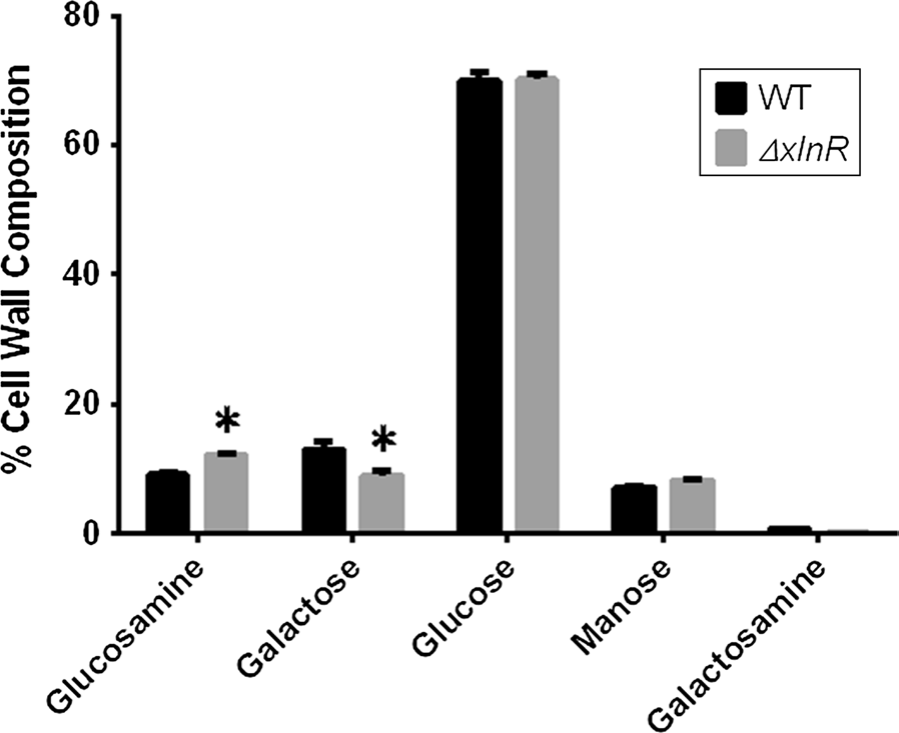
Cell wall analysis of sugars in the cell wall of the WT and *ΔxlnR* strains using HPLC

An analysis of the sugars in the cell wall of the WT and *ΔxlnR* strains was done using HPLC on the glucose-grown samples. The results showed significant differences, based on statistical analysis, in the contents of glucosamine and galactose with the *ΔxlnR* strain containing around 3 % more glucosamine and around 4 % more galactose than the WT strain (Fig. 6). There were no significant differences for the contents of glucose, mannose and galactosamine between the two strains.

### The cell wall of the *ΔxlnR* and *ΔclrB* strains is more permeable and the alternative oxidase pathway is up-regulated in the both strains

**Fig. 7:**
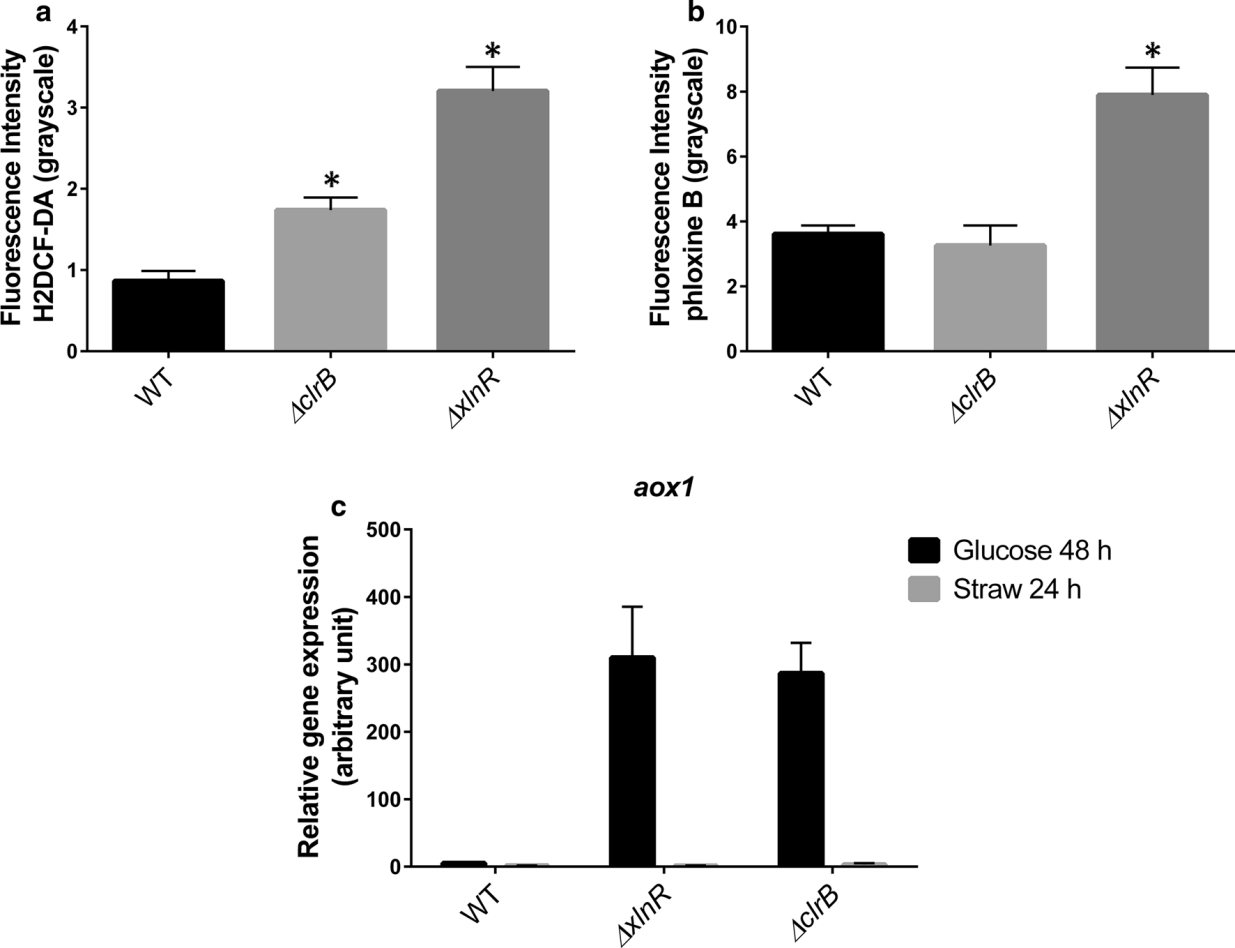
Reactive oxygen species (ROS) accumulation and cell permeability using the dyes H2DCF-DA (**a**) and phloxine B (**b**). Phloxine B stains the cytosolic material due to a more permeable cell in the *ΔxlnR* and *ΔclrB* strains. H2DCF-DA emits a green fluorescence when it is oxidised by ROS in the cell. The fluorescence intensity was measured using the ImageJ software. The *results shown* represent 50 measurements of three independent biological samples and the *error bars* indicate the standard error of the mean. * represents the conditions for which the *t*-test result had a *p*-value < 0.05 which means that the difference observed is significant when compared to the WT. **c** Expression of the alternative oxidase gene (*aox1*) measured by qRT-PCR in the WT and the *ΔxlnR* and *ΔclrB* strains. The *results shown* represent triplicates of three independent biological samples and the *error bars* indicate the standard error of the mean

The cell permeability of the *xlnR* deletion strain was assessed using the dye Phloxine B. This dye stains cytosolic material due to a loss of the integrity of the cell making it more permeable to Phloxine B. The fluorescence intensity was measured for 50 images for each strain in three biological repeats. The average intensity measured for the *clrB* deletion strain was around the WT one whereas the fluorescence intensity of the *xlnR* deletion strain was increased by 2.2 times in the deletion strain when compared to the WT intensity. An increase of the fluorescence means that more dye has entered the cell. The accumulation of Reactive Oxygen Species (ROS) was assessed using the fluorescent dyes 2′,7′-dichlorodihydrofluorescein diacetate (H2DCF-DA). H2DCF-DA passively diffuses into cells and is retained intracellularly after cleavage by intracellular esterases. Upon oxidation by ROS, the non-fluorescent H2DCFDA is converted to the highly fluorescent 2′,7′-dichlorofluorescein (DCF). The fluorescence intensity was measured for 50 images for each strain in three biological repeats. The average intensity measured for the WT strain was 0.87, 1.74 for the *ΔclrB* strain and 3.2 for the *ΔxlnR* strain suggesting that the *ΔxlnR* strain may be exposed to an oxidative stress with an accumulation of intracellular ROS in the fungal cells (Fig. 7).

## Discussion

Genes that encode enzymes active towards polysaccharides (CAZy) are one of the major groups of genes induced in response to lignocellulose. Transcription factors that function as repressors and activators regulate the expression of these CAZy enzyme-encoding genes. One of the major TFs, considered a master regulator, involved in regulation of CAZy enzyme-encoding genes in response to lignocellulose is a binuclear zinc finger protein named XlnR (xylanase regulator) in *Aspergillus* spp. and XYR1 (xylanase regulator 1) in *T. reesei* and related species. XlnR/XYR1 are the key activators in *Aspergillus* spp. and *T. reesei* of genes encoding cellulase and hemicellulase enzymes (Stricker et al. 2006; 2008; van Peij et al. 1998a; 1998b). In *N. crassa*, in contrast, the *xlnR/xyr1* orthologue *xlr*-*1* does not have the same function in regulating expression of cellulase-encoding genes as in *A. niger* or *T. reesei*. In the *Δxlr*-*1* mutant, growth on cellulose, and secreted cellulolytic activity, are only slightly affected compared to the WT (Sun et al. 2012). Instead, two other Zn_2_Cys_6_ family TFs CLR-1 and CLR-2 were found to be the predominant regulators of the expression of cellulase-encoding genes in *N. crassa* (Coradetti et al. 2012). Coradetti et al. (2012) demonstrated that these two TFs are important regulators of genes encoding both cellulases and hemicellulases in the presence of cellulose (Avicel) as carbon source, but they are not required for growth or hemicellulase production in the presence of xylan. In *A. oryzae*, the orthologue of CLR-2 called ManR was characterised as a regulator of genes encoding mannan-degrading enzymes (Ogawa et al. 2013).

In *A. niger*, the roles of ClrB in the *Aspergilli* are distinct from the roles in *N. crassa*. When comparing the roles of the *clr*-*2*/*clr*-*B* homologues in both *N. crassa* and *A. nidulans*, Coradetti et al. (2012) showed that the induction level of major cellulase-encoding genes in the *Δclr*-*B* deletion strain was several thousand-fold less than WT on Avicel, but in the *Δclr*-*A* deletion strain, the transcript level of the studied genes was two- to four-fold less. In our results, the same induction pattern was observed for both the *eglC* and the *xynA* genes. The *clrA* deletion strain showed a 3-fold decrease in its transcript level for the *eglC* gene and a similar transcript level for the *xynA* gene when compared to the WT. This suggests that, as in *A. nidulans* for which the induction of cellulase genes required *clr*-*B* but not *clr*-*A* (Coradetti et al. 2012), the *A. niger clrA* homologue may not be involved in the induction of GH-encoding genes. However, for all the genes tested, the transcript level of the *ΔclrB* deletion strain was significantly decreased when compared to the WT strain showing similarities with the induction patterns observed in *A. nidulans* (Coradetti et al. 2012). Nonetheless, a recent study showed that differences in the role of XlnR amongst *Aspergillus* species existed (Kowalczyk et al. 2015) which could also be the case for ClrA and ClrB.

The CAZy enzymes active upon wheat straw are secreted by the fungus to degrade wheat straw and release carbon sources and inducers that would aid its growth. A lack of induction of the enzymes essential for growth on the substrate would result in an impaired growth of the fungus as well as less enzyme activity from the supernatants of the cultures grown on wheat straw. A wide range of acids (e.g. citric acid, gluconic acid) is produced by *A. niger* during fermentation. Cultivation of *A. niger* can convert as much as 95 % of available carbon to organic acids (Karaffa and Kubicek 2003). Secretion of acids helps to degrade the plant cell walls on which the fungus grows, but it also slows the growth of competing organisms (Pedersen et al. 2000). Thus pH plays a very significant role in fungal growth and can be a good indicator of growth for *A. niger* (Archer et al. 1990). In our data, neither the *xlnR* nor the *clrB* deletion strains lowered the pH of the cultures in wheat straw as low as in the WT or the *ΔclrA* strains. The measurement of the glucosamine content of the cultures as a measure of growth showed that cultures of the *clrB* and *xlnR* deletion strains had lower glucosamine contents supporting the results from the pH measurements suggesting that these strains grew less well than the WT. The *xlnR* deletion strain is known to grow poorly on wheat straw (van Peij et al. 1998a). However, an increase of the glucosamine content over time was observed although it was less than for the WT strain. Nonetheless, the cell wall analysis undertaken revealed that the cell wall composition of the *ΔxlnR* strain was different to that of the WT strain with more glucosamine and less galactose in its cell wall. This may have influenced the result of the glucosamine assay and, therefore, not provide an accurate measure of growth of the *ΔxlnR* strain.

As the functioning of enzymes can also be influenced by the pH of the environment or the media, the *ΔxlnR* and *ΔclrB* strains were grown in a fermenter controlled at pH5.0 in order to confirm that the growth results, as well as the gene expression data, were the outcome of the lack of growth by the non-expression of the degrading enzymes rather than a pH effect. Our data showed that the transcript levels of *cbhA*, *eglC* and *xynA* were the same in the flask cultures as in the fermenter condition. This was especially noticeable for the *ΔxlnR* strain for which the pH was lower by 1.5 units in the fermenter (5.0) compared to the pH in the flasks (6.5). Therefore, the phenotypes observed were due to the deletion of the genes encoding XlnR and ClrB showing the importance of both TFs in the degradation of wheat straw in *A. niger*. To relate these transcriptomic results to enzymatic activity, the release of sugars from wheat straw was assayed. The measurement of sugar reducing ends released from the incubation of wheat straw culture filtrates with wheat straw substrate showed that lower enzyme activities were present in the supernatants of the cultures for the *ΔxlnR*, *ΔclAB* and *ΔclrB* strains with the *ΔxlnR* and *ΔclrB* strains being the least capable of releasing sugar reducing ends. Protein banding patterns of culture filtrates (Additional file 1: Fig. S1) showed that the profiles of the *clrA* deletion strain, from both the 24 and 48 h samples, were very similar to the WT one while the one from the *clrB* deletion strain showed fewer bands and the one from the *xlnR* deletion strain showed a completely different pattern with fainter bands.

There are significant differences in how XlnR in *A. niger* and XYR1 in *T. reesei* regulate transcription of their target genes (Stricker et al. 2008). In *T. reesei*, XYR1 interacts with the co-regulators ACE1 and ACE2 (activator of cellulase expression 2) (Aro et al. 2001) whereas *A. niger* lacks an *ace2* orthologue (Stricker et al. 2008; Todd et al. 2014). Furthermore, the *ace1* orthologue does not have the same function in *Aspergillus* spp. as shown by deletion of the *ace1* orthologue *stzA* in *A. nidulans* (Chilton et al. 2008). Chilton et al. (2008) showed that in *T. reesei* ACE1 was involved in cellulose utilisation but its orthologue in *A. nidulans*, StzA, controlled the abiotic stress response in this species. The qRT-PCR data showed that the *clrB* gene was more highly induced on wheat straw than the *clrA* gene. However, in the *xlnR* deletion strain, the transcript levels of *clrA* and *clrB* were decreased by 2.4 times and 4.8 times respectively when compared to the WT strain. This suggests that XlnR may regulate the two genes. In *N. crassa*, CLR-1 and CLR-2 interact for the induction of specific genes (Coradetti et al. 2012). In *A. niger*, *clrA* is not highly induced, and an interaction between ClrA and ClrB may not occur. Instead, another possibility would be that XlnR and ClrB interact together for the induction of a subset of genes. This hypothesis could be tested by doing a co-immunoprecipitation or a pull down assay. If XlnR and ClrB interact together, these methods would allow for the identification of their complex and of the targeted DNA sequence.

Kawasaki et al. (2002) showed that the High Osmolarity Glycerol (HOG) pathway was required for normal oxidative resistance in *A. nidulans* with the deletion of the *HOG1* homologue, *sakA*, resulting in a high sensitivity to oxidative stress. Few studies have associated oxidative stress with the induction of Alternative Oxidase (AOX) at the transcript and protein level (Morgan et al. 2008; Welchen et al. 2012). In our results, the gene *aox1* was highly induced in both the *clrB* and *xlnR* deletion strains. The use of the dye H2DCF-DA, as a marker of oxidative stress, indicated the accumulation of ROS for the two strains, suggesting that the *ΔclrB* and the *ΔxlnR* strains undergo an oxidative stress when grown on glucose. These results are supported by the transcriptomic data from de Souza et al. (2011) where several genes encoding proteins with oxidoreductase activity were found to be up-regulated in the *ΔxlnR* strain. In addition to being sensitive to oxidative stress, the *ΔxlnR* strain displayed cell wall defects with increased sensitivity to phloxine B although no altered sensitivity to SDS, Calcofluor White or Congo Red was found (Additional file 1: Fig. S3).

## Additional file

10.1186/s13568-016-0177-0 **Table S1**. Primers used in this study. **Fig S1**. Silver stained protein gel on supernatants from cultures transferred to wheat straw for 24h and 48h. **Fig S2**. Expression of the *actA* gene measured by qRTPCR in the WT, *ΔclrA*, *ΔclrB* and *ΔxlnR* strains. The results shown represent triplicates of three independent biological samples and the error bars indicate the standard error of the mean. **Fig S3**. Test for a cell wall stress by using cell wall stressors such as Sodium Dodecyl Sulfate (SDS, 70 μg/ml), CalcoFluor White (CFW, 50 μg/ml) and Congo Red (CR, 250 μg/ml) in Petri dishes.
