# Supplementary material for: The roles of the zinc finger transcription factors XlnR, ClrA and ClrB in the breakdown of lignocellulose by Aspergillus niger
Source: AMB Express. 2016 Jan 16;6:5. doi: 10.1186/s13568-016-0177-0 (PMC4715039; doi:10.1186/s13568-016-0177-0)
Supplement: Supplementary file 1 — 10.1186/s13568-016-0177-0 Table S1. Primers used in this study. Fig S1. Silver stained protein gel on supernatants from cultures transferred to wheat straw for 24h and 48h. Fig S2. Expression of the actA gene measured by qRTPCR in the WT, ΔclrA, ΔclrB and ΔxlnR strains. The results shown represent triplicates of three independent biological samples and the error bars indicate the standard error of the mean. Fig S3. Test for a cell wall stress by using cell wall stressors such as Sodium Dodecyl Sulfate (SDS, 70 μg/ml), CalcoFluor White (CFW, 50 μg/ml) and Congo Red (CR, 250 μg/ml) in Petri dishes. [file 13568_2016_177_MOESM1_ESM.pdf]

The roles of the zinc finger transcription factors XlnR, ClrA and ClrB in the breakdown of lignocellulose by *Aspergillus niger*

Applied Microbiology and Biotechnology Express

Roxane Raulo, Matthew Kokolski and David B. Archer
School of Life Sciences, University of Nottingham, University Park, NG7 2RD, Nottingham, UK

Corresponding author:
david.archer@nottingham.ac.uk
Tel: 0115 951 3313
Fax: 0115 951 3251

Additional file 1: **Table S1**. Primers used in this study. **Fig S1.** Silver stained protein gel on supernatants from cultures transferred to wheat straw for 24h and 48h. **Fig S2.** Expression of the*actA* gene measured by qRTPCR in the WT, *ΔclrA*, *ΔclrB* and *ΔxlnR* strains. The results shown represent triplicates of three independent biological samples and the error bars indicate the standard error of the mean. **Fig S3.** Test for a cell wall stress by using cell wall stressors such as Sodium Dodecyl Sulfate (SDS, 70 µg/ml), CalcoFluor White (CFW, 50 µg/ml) and Congo Red (CR, 250 µg/ml) in Petri dishes.

**Name**	**Sequence (5' - 3')**	**Function**
AnXlnRNotIup	ATGCATGCGGCCGCTCCCAACTTTATTCACTCC C	*xlnR* deletion upstream external
AnXlnRHindIIIup	ATGCATAAGCTTGGAATTTCGCAAGGAAGTGG	*xlnR* deletion upstream internal
AnXlnRHindIIIdw	ATGCATAAGCTTTTTTGCAGTAACACGGCTG	*xlnR* deletion downstream internal
AnXlnRspeIdw	ATGCATACTAGTACTCACGGGATCCCACGAAG	*xlnR* deletion downstream external
AnXlnRScexup	CAGACTGAATCGGCAATGC	Screen *xlnR* deletion, external
AnXlnRScexdw	GTTTAAAGGAGGGGGTTTGG	Screen *xlnR* deletion, external
AnXlnRScinup	CACCATCTGAGCTCGCAGCC	Screen *xlnR* deletion, internal
AnXlnRScindw	GCAGTTTCTGGCATACACG	Screen *xlnR* deletion, internal
Clr1 up F	tattatGAATTCACGCGGCTTTCAAACATATC	*clrA* deletion upstream external
Clr1 up R	tattatGGTACCCACCGAGGGCTGAAAGCATC	*clrA* deletion upstream internal

Clr1 down F	tattatGGTACCAAGAGGCACTTGACTTGCAG	*clrA* deletion downstream internal
Clr1 down R	tatatGCGGCCGCCGTCATTGTCCCGAGAAGAA	*clrA* deletion downstream external
Clr1 gene F	CCCTCTACAACCCTCACCAA	Screen *clrA* deletion, internal
Clr1 gene R	TGAGGCCTGGACGCTACTAT	Screen *clrA* deletion, internal
qPCR BglB F	AGGTGGAGATTGTGGTGGAG	qRT-PCR *bglB*
qPCR BglB R	CCCAATACCTCGTCCTCTCA	qRT-PCR *bglB*
qPCR ClrA F	GCCTCTCATTCGAGAACTGG	qRT-PCR *clrA*
qPCR ClrA R	CGTCAATGGGTTGACACAAG	qRT-PCR *clrA*
qPCR ClrB F	GGACGGCAATTGAAAGATGT	qRT-PCR *clrB*
qPCR ClrB R	AGCTGTCGCTCTTGGATCAT	qRT-PCR *clrB*
qPCR EglC	CGATGGTGTTACCGGTCTCT	qRT-PCR *eglC*
qPCR EglC R	GCCGAAGTCGAACTCTTCAC	qRT-PCR *eglC*
qPCR XynA	TTATGCCTGGGATGTTGTCA	qRT-PCR *xynA*
qPCR XynA R	TGCCGGTCAATTTAGGGTAG	qRT-PCR *xynA*
qPCR cbhA F	CCAGCAAGCCGGAACGCTCA	qRT-PCR *cbhA*
qPCR cbhA R	AACGCGCCGTTTAGCCCACA	qRT-PCR *cbhA*

Table S1 Primers used in this study

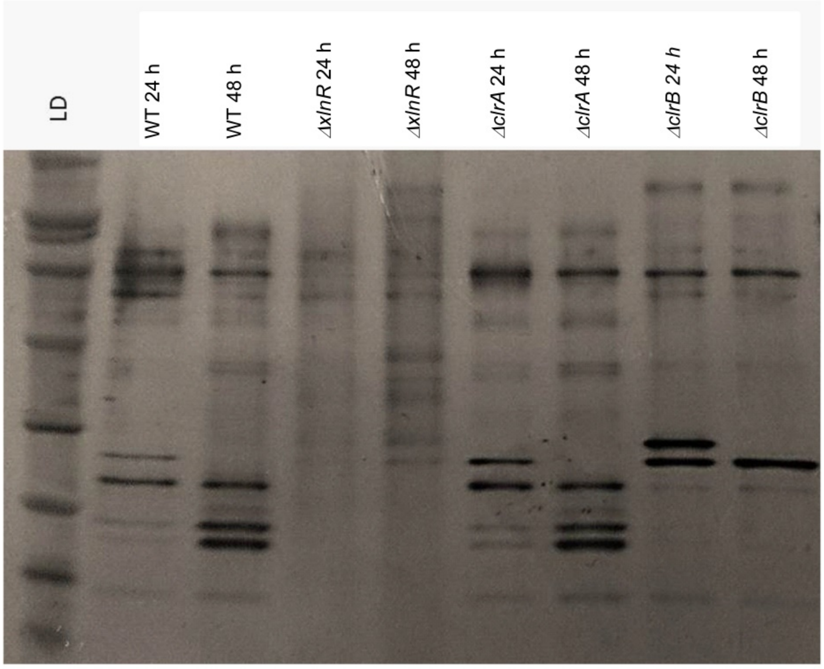

Fig.S1 Silver stained protein gel on supernatants from cultures transferred to wheat straw for 24 h and 48 h

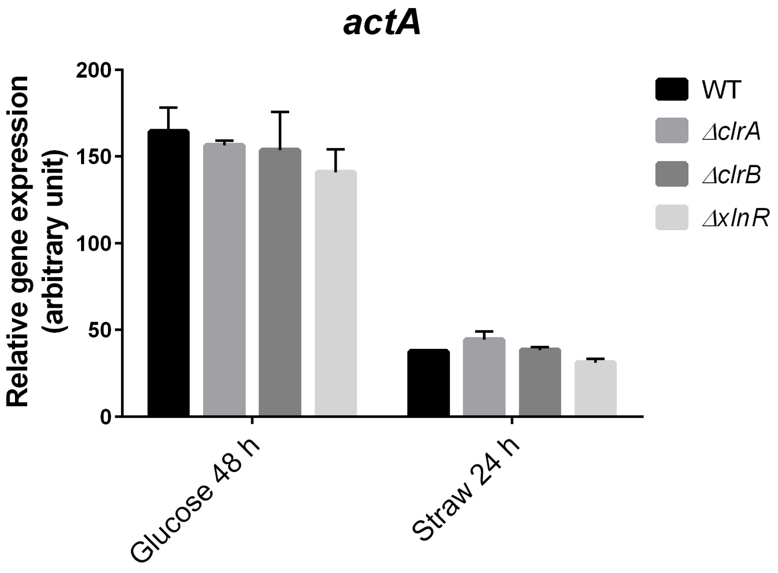

Fig.S2 Expression of the *actA* gene measured by qRTPCR in the WT, *ΔclrA*, *ΔclrB* and *ΔxlnR* strains. The results shown represent triplicates of three independent biological samples and the error bars indicate the standard error of the mean

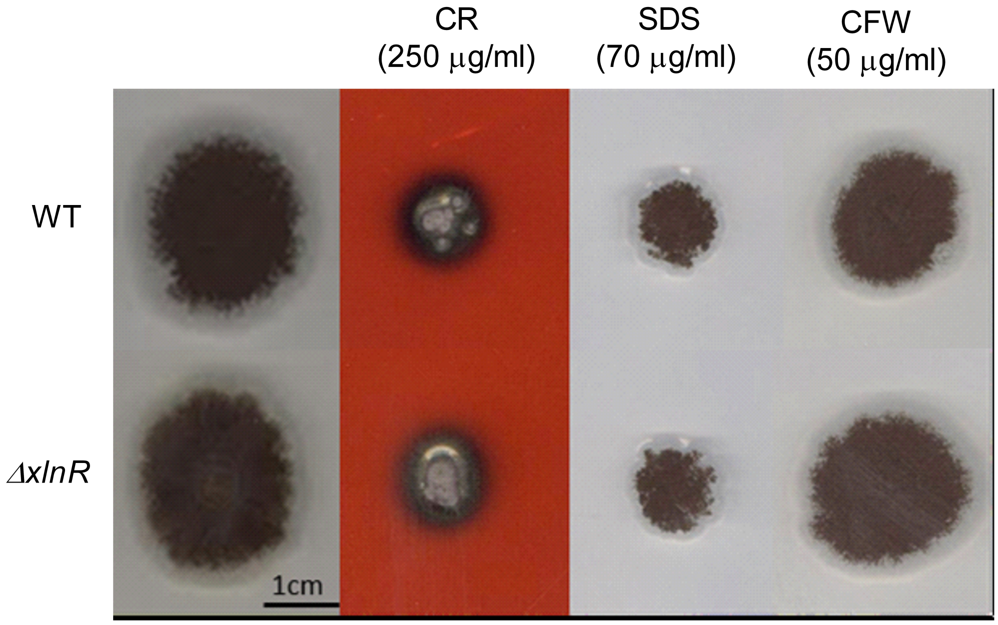

Fig.S3 Test for a cell wall stress by using cell wall stressors such as Sodium Dodecyl Sulfate (SDS, 70 μg/ml), CalcoFluor White (CFW, 50 μg/ml) and Congo Red (CR, 250 μg/ml) in Petri dishes
